# Laser-Enabled Surface Treatment of Disposable Endoscope Lens with Superior Antifouling and Optical Properties

**DOI:** 10.1021/acs.langmuir.2c01671

**Published:** 2022-09-07

**Authors:** Themistoklis Karkantonis, Anvesh Gaddam, Himani Sharma, Gerard Cummins, Tian Long See, Stefan Dimov

**Affiliations:** † Department of Mechanical Engineering, School of Engineering, The University of Birmingham, Birmingham B15 2TT, U.K.; ‡ Department of Chemical and Biomolecular Engineering, University of Notre Dame, Notre Dame, Indiana 46556, United States; § The Manufacturing Technology Centre Ltd., Coventry CV7 9JU, U.K.

## Abstract

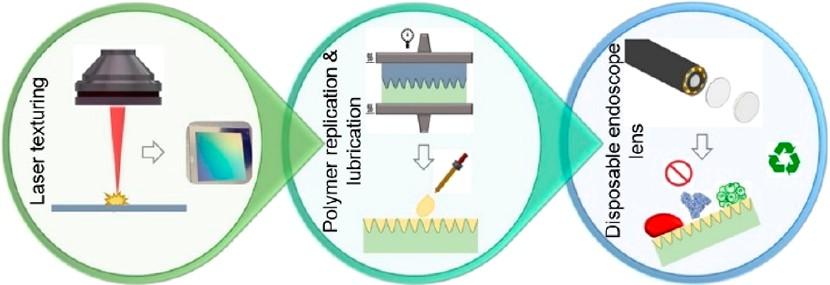

Endoscopes are ubiquitous in minimally invasive or keyhole surgeries globally. However, frequent removal of endoscopes from the patient’s body due to the lens contaminations results in undesirable consequences. Therefore, a cost-effective process chain to fabricate thermoplastic-based endoscope lenses with superior antifouling and optical properties is proposed in this research. Such multifunctional surface response was achieved by lubricant impregnation of nanostructures. Two types of topographies were produced by femtosecond laser processing of metallic molds, especially to produce single-tier laser-induced periodic surface structures (LIPSS) and two-tier multiscale structures (MS). Then, these two LIPSS and MS masters were used to replicate them onto two thermoplastic substrates, namely polycarbonate and cyclic olefin copolymer, by using hot embossing. Finally, the LIPSS and MS surfaces of the replicas were infiltrated by silicone oils to prepare lubricant-impregnated surfaces (LIS). Droplet sliding tests revealed that the durability of the as-prepared LIS improved with the increase of the lubricant viscosity. Moreover, the single-tier LIPSS replicas exhibited longer-lasting lubricant conservation properties than the MS ones. Also, LIPSS-LIS replicas demonstrated an excellent optical transparency, better than the MS-LIS ones, and almost match the performance of the reference polished ones. Furthermore, the LIPSS-LIS treatment led to superior antifouling characteristics, i.e., regarding fogging, blood adhesion, protein adsorption, and microalgae attachment, and thus demonstrated its high suitability for treating endoscopic lenses. Finally, a proof-of-concept LIPSS-LIS treatment of endoscope lenses was conducted that confirmed their superior multifunctional response.

## 1 Introduction

In recent years, minimally invasive medical operations such as laparoscopy and endoscopy have attracted considerable attention among surgeons, as they are relatively quick and safe procedures to treat patients. Because such keyhole surgeries require small incisions, less blood loss is associated with them and also the infection risks are less, while the postsurgery recovery time of the patients is much shorter.^1,2^ For instance, endoscopes have been deployed widely to probe organs for cancer, collect biopsies from tissues, and deliver treatments inside the human body. The principal procedures and overall performance of these contemporary techniques are strongly dependent on visualization capabilities of optical devices, such as endoscopes, and thus their field of view must be maintained intact throughout these surgical interventions. However, the endoscope lenses can be contaminated/fouled by blood and other body fluids, i.e., fluids that contain fats and proteins, or even become foggy due to some condensations during endoscopic surgeries.^3,4^ In general, these substances can adhere to the lens surfaces, resulting in impaired vision and hence low efficiency of such surgical interventions.^5^ Therefore, there is a pressing need to design and manufacture endoscope lenses with enhanced antifouling functionalities without sacrificing their optical transparency under harsh surgical conditions.

To date, the most common methods for removing any impurities from the endoscope lenses in vivo surgeries involve manual wiping, i.e., the rubbing of the lens against adjacent tissues/organs or the extraction of the endoscope from the human body to wipe it off, and also the integration of extra working channels for irrigation and suction.^6^ Although the clarity of endoscopic vision can be retained to a certain extent by using these techniques, their practical implications might lead to internal organ injuries, surgical site infections, extended operation times due to unnecessary disruptions, and patient discomfort.^7^

To address such challenging requirements, various surface treatment methods have been deployed to tailor the functional responses of materials by modifying their surface topography and/or chemistry.^8,9^ Coating-based methods were proposed for creating hydrophilic surfaces with dual-functional antifogging/antibacterial response for optical devices.^10−12^ Especially, a stable liquid film on the surface can be formed by applying such coatings that obstructs the creation of scattering droplets and thus inhibits fogginess and maintains transparency in humid environments. Nevertheless, without applying any aftertreatment, e.g., rinsing with water, nontransparent liquids can spread and eventually adhere to such hydrophilic surfaces, and therefore they are prone to contaminations and vision losses during endoscopic surgeries.

Superhydrophobic surfaces have received great attention, too, from researchers because of their excellent antiwetting,^13^ anti-icing,^14^ antibiofouling,^15^ antifriction,^16^ and antibacterial^17^ responses. Specifically, the use of a low surface energy coating, e.g., perfluorinated compounds, and hierarchical micro-/nanoscale topographies to entrap air were applied to achieve robust superhydrophobic and/or superoleophobic behaviors.^18^ The heterogeneous wetting state, i.e., Cassie–Baxter state (CBS), on such surfaces can exhibit an extreme repellency even against complex fluids like blood.^19,20^ However, CBS is metastable, especially upon exposure to low surface tension liquids^21^ and moderate pressure,^22^ and a transition to the Wenzel state can completely destroy the attractive wetting properties of such surfaces. In addition, a relatively high surface roughness is beneficial for their superhydrophobic properties, while impacting negatively their transparency. Therefore, attaining both functionalities is technically challenging and hence a time-consuming task^23,24^ that together with the limitations of such treatments can render them inadequate for fulfilling the requirements of medical optic devices.

At the same time, lubricant impregnated surfaces (LIS) have gained significant interest as a way to address the aforementioned shortcomings, especially by impregnating micro/nano-structured surfaces with a low-energy lubricant. Specifically, LIS can offer excellent long-lasting liquid shedding characteristics, i.e., display very low critical sliding angle (CSA) even against yield stress fluids,^25^ stability under high-pressure and -temperature conditions,^26^ and antibiofouling/antibacterial/antithrombogenic/drag-reduction properties.^27−29^ In addition, numerous studies have demonstrated that LIS with such functional characteristics can be prepared with coating techniques without compromising the optical properties of transparent materials.^30−35^

Because of these advantages, LIS have emerged as a very promising option for improving the efficacy of endoscopic surgical procedures by keeping the camera lens clear from foulants. For example, two relatively similar treatments have been proposed to produce antibiofouling materials for endoscope lenses by depositing silica nanoparticles onto glass substrates as a base porous structure and then lubricating it with either silicone or plant oil.^36,37^ Even though both materials were capable of maintaining a clear endoscopic vision by repelling blood and body fluids repeatedly, the employed surface treatment methods did not meet the requirements for their scale-up production. In this regard, Tenjimbayashi et al.^38^ reported a simple one-step procedure to produce a lubricated fiber-filled porous material, which can be synthesized in situ and then adhered to the endoscope lens for single use. Nevertheless, the nanofibrous LIS proposed in this research raised significant concerns about their mechanical durability and chemical stability. To overcome the intrinsic limitations associated with the coating-based techniques, Lee et al.^39^ proposed an alternative surface treatment to functionalize endoscopic lenses by direct laser texturing. Prior to lubricant infusion, only a fluorinated self-assembled monolayer was applied to the textured surface and thus to enhance the longevity of its antibiofouling performance. While highly ordered structures could be produced selectively by laser surface texturing even onto freeform surfaces with high precision, the high operating costs associated with this stand-alone process hamper its broader application. At the same time, the use of coatings that contain long chain perfluorinated compounds for preparing LIS can have a negative impact on both human health and the environment.^40,41^ Therefore, further research is necessary to address the issue of vision loss in endoscopic surgeries by developing a cost-effective process chain for producing optical lenses with a multifunctional response. Also, the environmental impact of any method for their scale-up manufacture should be considered, especially they should be recyclable without requiring any aftertreatments.

This research reports an efficient and cost-effective process chain for producing recyclable disposable endoscopic lenses with superior durability while exhibiting excellent antifouling properties and transparency. The method that includes, first, the manufacture of metallic masters with both submicrometer and multiscale topographies, then replicating them on thermoplastic substrates, and finally impregnating the replicas with silicone oil is described. Next, the optical and antiadhesive properties of produced LIS together with their lubricant retention capabilities were investigated under shear flow conditions. To demonstrate the effectiveness of such lens treatment for preventing vision loss during endoscopy, LIS were further subjected to protein adsorption, microalgae adhesion, blood fouling, and fogging tests. Finally, a set of LIS lenses was integrated into an inspection endoscope device to demonstrate the proof of concept.

## 2 Materials and Methods

**Figure 1:**
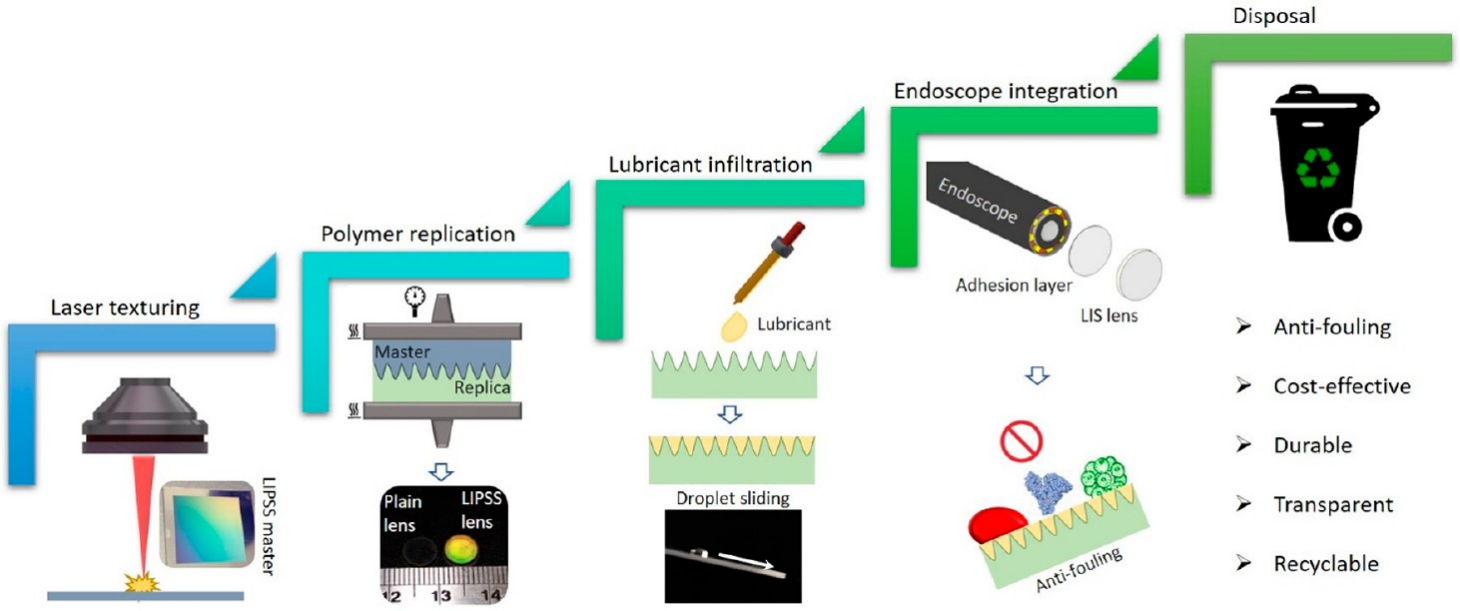
Overview of the process chain employed to fabricate disposable endoscope lenses.

An overview of the proposed process chain for producing disposable and recyclable endoscopic polymer lenses that exhibit multifunctional responses is shown in Figure 1. The employed processing steps in this chain together with the respective methods used to characterize the surface of produced lenses are described in this section.

### 2.1 Fabrication of Metallic Masters

Commercially available AISI 316 stainless steel (SS) plates with a surface area of 40 × 40 mm^2^ and a thickness of 0.5 mm were used as metallic masters. The as-received substrates had an initial average surface roughness (Sa) of 35 nm and were then laser treated. The surface texturing was performed on a laser processing workstation (LASEA LS5, Belgium) under atmospheric conditions. In particular, the system integrates a femtosecond Yb-doped laser source (Satsuma, Amplitude Systems) with the following technical specification: a pulse duration of 310 fs, nominal wavelength of 1030 nm, pulse repetition rate (*f*) up to 500 kHz, and maximum average power of 5 W. In addition, a half-waveplate was incorporated into the beam delivery subsystem to texture the SS substrates with an s-polarized Gaussian beam. Thereafter, a galvo scan head was employed to deflect the focused laser beam across the samples at a maximum scanning speed (*v*) of 2000 mm/s, and a 100 mm telecentric lens was used to focus the beam to a spot size of 40 μm at 1/e^2^ of the Gaussian intensity. Finally, a high precision stack of three linear motorized stages (*X*, *Y*, *Z*) in combination with a high-resolution camera was utilized for positioning samples with high accuracy and repeatability prior to the laser surface texturing operations.

Two different surface topographies, namely, single-tier laser-induced periodic surface structures (LIPSS), i.e., nanoscale ripplelike structures, and two-tier multiscale structures (MS), i.e., microscale protrusions with nanoscale ripples on top of them, were produced onto the SS substrates, following the approach reported in another research.^42^ Briefly, MS had a gridlike microtopography that was produced employing the following process settings: a fixed *f* of 500 kHz, a *v* of 1000 mm/s, and a constant hatch (*h*) distance of 40 μm between any two consecutive lines in *X* and *Y*. In total, 40 scans, i.e., 20 scans per line, with a single pulse fluence (φ_0_) of 178 mJ/cm^2^ were required to produce a relatively shallow grid of microprotrusions on the SS surface. At the same time, the single-tier LIPSS were generated by employing a raster scanning strategy and a single pass over the surface with a fixed *f* of 250 kHz, a *v* of 1000 mm/s, an *h* of 3 μm, and the same φ_0_. The laser parameters used for creating the two surface topographies investigated in this research together with the overall processing time required to complete their texturing operations over an area of 30 × 30 mm^2^ are summarized in Table 1.

**Table 1:** Key Laser Parameters Used to Produce the MS and LIPSS onto the SS Molds Together with Their Corresponding Processing Time

topography	*f* (kHz)	*v* (mm/s)	φ_0_ (mJ/cm^2^)	*h* (μm)	total no. of scans	processing time (min)
MS	500	1000	178	40	40	15
LIPSS	250	1000	178	3	1	4

### 2.2 Fabrication of Lubricant Impregnated Polymer Lenses

Single-tier LIPSS and two-tier MS textures on SS surfaces were replicated onto commercially available transparent polycarbonate (PC) and cyclic olefin (COC) polymer sheets with a thickness of 1.5 mm (purchased from Microfluidic ChipShop, Germany) by using a hot embossing system. These thermoplastics are commonly used for optical lenses and can withstand autoclave temperatures. The glass transition temperatures of these polymers as received are given in Table 2. During the embossing process, the textured SS molds together with the as-received polymer sheets were pressed to each other at an elevated temperature, i.e., higher than their glass transition temperatures. Because the replication quality was strongly affected by the applied temperature and load, the influence of these two parameters was investigated to identify the optimum processing window for both polymer substrates. The process settings used to produce the PC and COC replicas are provided in Table 2.

**Table 2:** Optimized Settings Used to Produce the PC and COC Replicas Together with Their Respective Glass Transition Temperatures

polymer	load (kN)	operating temp (°C)	glass transition temp (°C)	compression time (min)
PS	9.2	150	145	10
COC	10.8	145	142	10

Lastly, silicone oils (Sigma-Aldrich) with different viscosities, i.e., 5, 20, and 100 cSt, were used as lubricants to prepare LIS in this study. After the lubricant oil was pipetted and completely spread onto the entire surface of the textured replicas due to capillary forces, the samples were kept vertically for an hour to drain any excess oil. Compared to other types of lubricants, e.g., perfluorinated oils, the rationale for selecting silicone-based ones for preparing LIS in this research is associated with the fact that they are nontoxic to the human body, and also their use has been approved by both FDA^43^ and EFSA^44^ for medical applications as well as food additives. Therefore, the as-prepared thermoplastic LIS can be considered of being biocompatible and thus suitable for the usage as objective lenses in endoscopes.

### 2.3 Morphology and Optical Characterization

The LIPSS and MS topographies produced on the SS and then replicated on the thermoplastic substrates were initially inspected by using a scanning electron microscope (SEM, Hitachi TEM3030Plus). The dimensional characteristics of the MS structure were obtained by using a focus variation optical microscope (Alicona G5), while an atomic force microscope (AFM, MFP-3D, Asylum Research) was employed to study the morphology of the nanoscale surface topographies. An open-source image analysis software (Gwyddion) was used to process the acquired 10 × 10 μm^2^ AFM scans and thus to obtain the LIPSS cross-sectional profiles. At the same time, this software was also utilized to gain information about their orientation and spatial periodicity by performing a 2D-FFT analysis. The transmittance of the as-received, textured, and lubricated thermoplastic surfaces was analyzed over the visible spectrum, i.e., from 400 to 700 nm, employing a UV–vis spectrometer (Lambda 365, PerkinElmer). To ensure the reliability and validity of these results, the transmittance measurements were conducted in triplicates.

### 2.4 Wettability Characterization

The wettability of the as-received, textured, and lubricated thermoplastic substrates was investigated by using the sessile drop technique. Specifically, 5 μL water droplets were dispensed onto these surfaces, and then their static contact angle (CA) values were measured with a goniometer (OCA 15EC, Data Physics GmbH, Germany). Also, the contact angle hysteresis (CAH) of the aforesaid surface topographies was analyzed by gradually increasing and decreasing the volume of the water droplet on the surface, and the difference between the advancing and receding contact angles was quantified. At the same time, the shedding characteristics of the thermoplastic plain, textured, and LIS samples were examined, too. First, the critical sliding angle (CSA) values of 10 μL water droplets were measured on these surfaces by employing a motorized tilting stage with positioning accuracy and resolution of 10 arcsec and 0.01°, respectively. Thereafter, CSA of other liquids such as Xanthan gum solution and pH liquids was investigated to simulate the response of LIS against viscous fluid substances, e.g., mucus, body fluids, and gastric fluids with different pH levels. The concentration of Xanthan gum in the aqueous solution was 2 g/L, while the preparation procedure and the shear viscosity properties of this liquid were analytically described in our previous study.^45^ Finally, it should be stated that the average CA, CAH, and CSA values reported in this study were calculated based on five repeated measurements taken at different locations on each sample.

### 2.5 Durability Characterization

Generally, the LIS performance is expected to degrade when body fluids start wetting continuously their surfaces. Sooner or later, this will allow contaminants to foul the camera lens and obstruct vision during endoscopic surgeries. The reason for the loss of the LIS functionality can be mainly attributed to the depletion of impregnated lubricant under persistent shear flow conditions.^46^ To assess the LIS durability, LIS prepared with the silicone oils of varying viscosity were tilted at 15° by using a motorized stage, while 10 μL water droplets were continuously dispensed on their surfaces up to the point where no motion could be detected; i.e., the droplet was completely pinned to the surface. To ensure that the droplets flowed at exactly the same spot and also to avoid them bouncing on the surface during the tests, a fixture was used to hold the pipet above the substrates and release the droplets from a maximum height of 2 mm. A camera (Canon 2000D) mounted on a tripod was employed to record the mobility of water droplets at 60 frames/s. Lastly, an open-source video analysis and modeling software (Tracker) was used to obtain the steady-state shedding velocity of the water droplets.

### 2.6 Antifouling Characterization

To characterize antifouling capabilities of LIS on the embossed thermoplastic substrates, three different tests were conducted with different foulants, as expected in potential endoscopic procedures, i.e., blood, protein, and microorganisms. First, the experiments were conducted to examine the antiadhesive ability of LIS against defibrinated sheep blood (Darwin Biological, UK). In particular, the mobility of single blood droplets was monitored on LIS when tilted at an angle of ∼45°. Thereafter, their blood-shedding capabilities were tested by immersing them into containers filled with blood for up to 30 times.

Next, LIS were assessed for their protein adsorption. For this, the thermoplastic plain and LIS substrates were incubated overnight with a solution of CFSE (carboxyfluorescein succinimidyl ester, λ_excitation_: 492 nm; λ_emission_: 517 nm) dye (ThermoFisher Scientifics, USA) tagged bovine serum albumin (Sigma-Aldrich, USA) (1% (w/v)) in a Petri dish under a dark environment. Subsequently, the plain and LIS substrates of both PC and COC were imaged by using a CCD camera (Retiga EXi, QImaging) connected to an inverted fluorescence microscope (Olympus IX71) with a 4× magnification lens. Both the bright field and the fluorescence images (Semrock LF488/561 green filter cube) of PC and COC were acquired with the microscope and were later merged into a composite image by using ImageJ.

Finally, the *Chlorella vulgaris* (CV) strain CCAP 211/11B (Darwin Biological, UK) was chosen for the assessment of microalgae adhesion to LIS. To this end, a preculture of CV along with the modified Bold’s basal medium was added to the glass containers, and the culture was incubated at 28 °C for a week under the cool white fluorescent illumination with a light-to-dark ratio of 12:12 h. The plain and LIS substrates of both PC and COC were placed inside the glass containers during the incubation. After a week, the substrates were imaged with a custom-made setup by taking advantage of the CV autofluorescence. To capture the fluorescence from the CV-settled substrates, a camera (Canon 2000D) fitted with an 18–55 mm lens and yellow and magenta filters was used. These fillers were employed to filter out the undesired wavelengths, apart from 680 nm red light, under the ultraviolet light excitation of the chlorophyll. In addition, a dioptre lens was also attached to the camera to obtain the magnified images. The obtained fluorescent images from the substrates were analyzed by using the ImageJ software to calculate the area covered by CV in percentages.

### 2.7 Proof-of-Concept Demonstration

Finally, to demonstrate the applicability of these surfaces for preventing blood fouling during endoscopy, the LIS lenses were integrated into a representative inspection endoscope device as follows: (i) a round flat lens of 8 mm in diameter was cut out of the textured polymer replica, (ii) the lens was thoroughly cleaned with isopropyl alcohol and lubricated as described in subsection 2.2, and (iii) an adhesion layer (Glue Dots) was used to attach it onto the endoscope camera. In this research, the visibility through the LIS lenses was verified by directing the endoscope onto a printed QR code and then trying to read it with a smartphone camera. In addition, their resistance against blood fouling was assessed by immersing/dipping the endoscope into blood multiple times until its vision was totally lost; i.e., the treated lens was fouled by blood. It should be stated that this blood fouling test was repeated for three identical LIS lenses. During these experiments, the integrated endoscope camera was employed to capture images of a printed chessboard. Thereafter, they were postprocessed with an open-source image analysis software (ImageJ) to quantify the percentage of the area visible after a certain number of dips.

## 3 Results and Discussion

### 3.1 Surface Morphology Analysis

**Figure 2:**
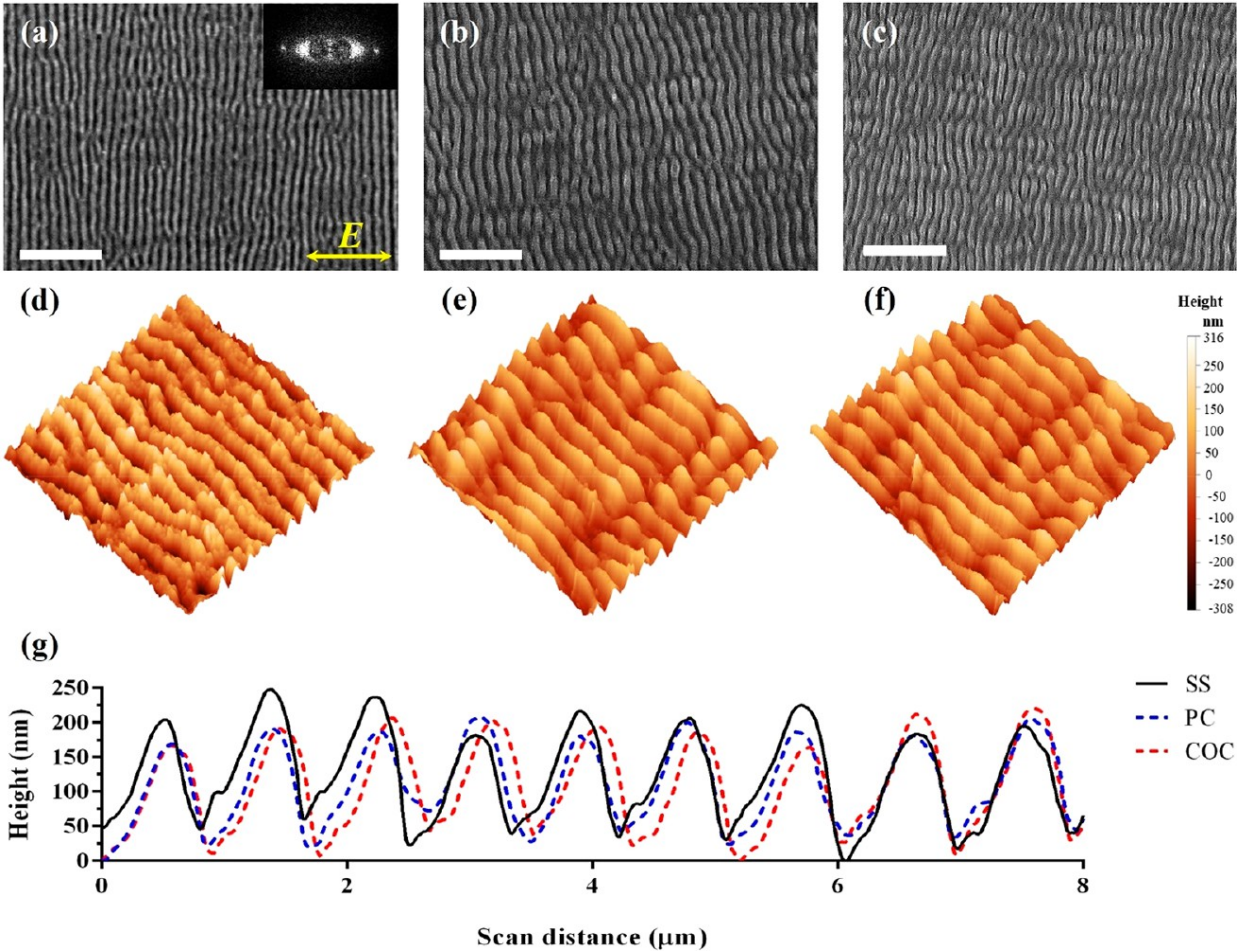
Surface morphologies of LIPSS: (a–c) SEM and (d–f) 3D AFM micrographs of LIPSS on the SS, PC, and COC surfaces, respectively. (g) Plot showing the surface profiles of LIPSS on the SS master and PC and COC replicas. Scale bar: 6 μm.

The first part of this study was focused on generating LIPSS and MS over an area of 30 × 30 mm^2^ on the SS substrates. Figure 2a shows a SEM micrograph of LIPSS generated on the surface when the SS master was irradiated with the femtosecond pulsed laser. As can be seen, the surface topography obtained represents uniform periodic ripplelike structures with an orientation perpendicular to the laser beam polarization vector (indicated by yellow double arrows in Figure 2a). From the 2D-FFT analysis performed at different areas on the textured SS surface, the main LIPSS spatial periodicity was slightly below the laser wavelength of 1030 nm and varied in the range from 800 to 900 nm. At the same time, the well-defined linear 2D-FFT image (see the inset of Figure 2a) can be considered as proof of the LIPSS regularity on the SS surface. After the hot embossing process, the LIPSS topography was successfully replicated onto the thermoplastic substrates. SEM micrographs of the LIPSS embossed onto the PC and COC substrates are depicted in Figures 2b and 2c, respectively. The 3D AFM images of LIPSS on the SS master and PC and COC substrates demonstrate the replication quality achieved, and they are given in Figure 2d–f, respectively. In addition, Figure 2g shows their respective surface profiles over a scan distance of 8 μm. These cross-sectional profiles were obtained based on the average values of scans taken at five different locations on the textured SS master and its polymer replicas. On the basis of the AFM profiles, the LIPSS average height was calculated to be within the range of 152–172 nm on all substrates, and this signifies an excellent replication quality. More specifically, the deviation in percentages from the average LIPSS height was less than 11 and 5%, when the average values obtained on PC and COC substrates were compared with that on the SS master, respectively.

**Figure 3:**
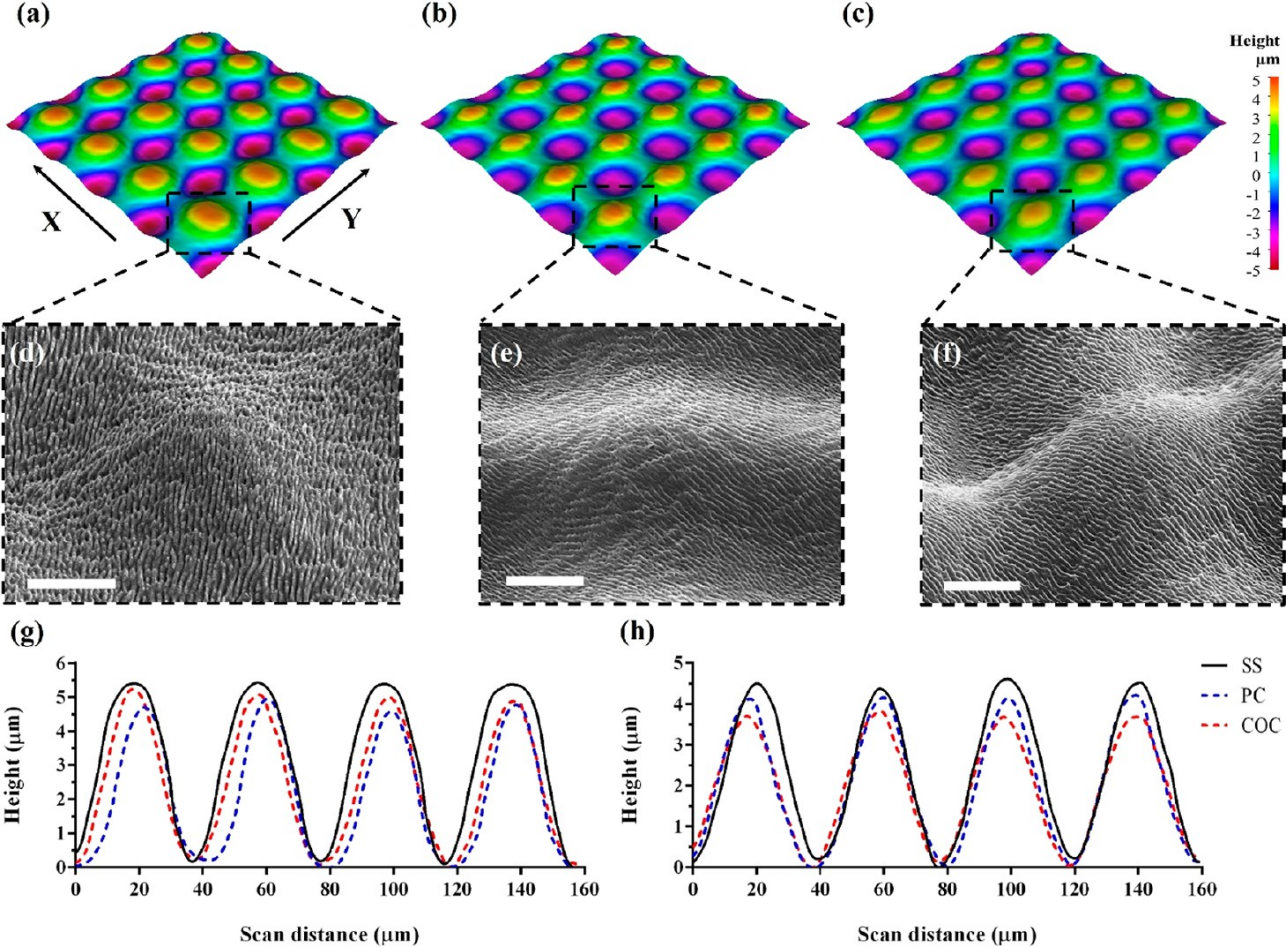
Surface morphologies of MS: (a–c) the 3D height map and (d–f) the SEM images of MS on the SS master and PC and COC substrates, respectively. (g, h) Plots showing the height profiles of micropeaks on the SS master and PC and COC substrates along *X* and *Y*, respectively. Scale bar: 20 μm.

Next, the MS topographies were produced on a SS master. Because of the higher fluence accumulated at the intersecting points of the perpendicular beam vectors in the gridlike scanning strategy, “peaks” and “valleys” were formed on the SS surface as shown in Figure 3a. By taking a closer look at Figure 3d, it is apparent that the microtopography is fully covered with nanoscale ripplelike structures. In reality, the dimensional and geometrical characteristics of these ripples should be almost identical with that of LIPSS. Following the embossing step, the negative patterns of the MS on the SS surface were replicated onto the PC and COC substrates as depicted in Figure 3b,c. At the same time, it is evident from Figure 3e,f that the nanoscale morphology on top of the peaks and valleys was replicated successfully onto the polymer substrates. To analyze further the replication quality achieved during the embossing process, the height profiles of micropeaks on the SS master and the PC and COC samples along *X* and *Y* were captured as shown in Figures 3g and 3h, respectively. No significant discrepancies were detected in the peak spacings onto the SS and polymer surfaces; i.e., they were consistent close to 40 μm on all of them. However, some relatively small variations can be observed in their heights across the substrates. For instance, the average height of micropeaks on the SS surface was 5.1 and 4.2 μm when measured at five different locations along *X* and *Y*, respectively, whereas the same measurement procedure led to average heights of 4.8 and 3.9 μm on PC and 4.9 and 3.6 μm on COC along *X* and *Y*, respectively. Overall, it can be stated that the difference of thermoplastic replicas from their metallic masters was less than 14%.

### 3.2 Wettability Analysis

**Figure 4:**
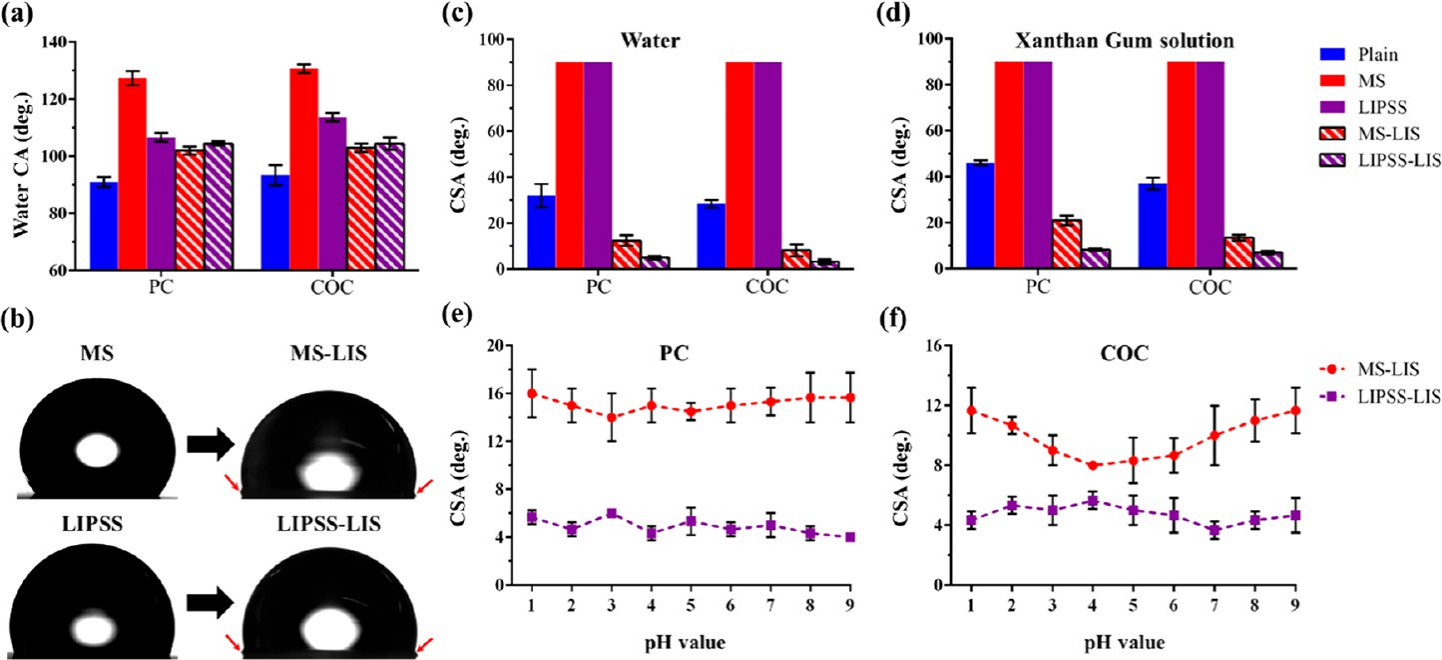
Wettability analysis: (a) the static CAs measured on as-received, textured, and lubricated surfaces of PC and COC substrates; (b) representative images of the water droplet on textured and lubricated surfaces of the COC substrate; the CSAs obtained on as-received, textured, and lubricated surfaces of the PC and COC substrates for (c) water and (d) Xanthan gum solution; the CSAs obtained on the lubricated surfaces of (e) PC and (f) COC.

An important part of this research was focused on characterizing the wettability of all thermoplastic functionalized surfaces, while using as a reference untreated ones. The static CAs obtained on as-received (plain), textured (MS, LIPSS), and lubricated (MS-LIS, LIPSS-LIS) surfaces of PC and COC substrates are given in Figure 4a. Initially, the plain surfaces of both thermoplastic materials exhibited a slightly hydrophobic behavior with average CA values just above 90°. However, CAs increased substantially when the laser textured SS masters were embossed onto them. As expected, the surfaces covered with MS displayed higher CA values on both PC and COC replicas compared to those with single-tier LIPSS. For instance, the average CA value measured on the MS of COC replica was 131°, while the single-tier LIPSS embossed on the same material had CA of 114°. A 5 μL water droplet led to a larger CA on MS than that on LIPSS, as depicted in Figure 4b. After the infusion of lubricant with 20 cSt viscosity onto the textured polymer substrates, the apparent CA decreased, especially on the MS-LIS samples, and a small wetting ridge appeared at the periphery of the hemispherical water droplets on all LIS (see the red arrows in Figure 4b). The formation of such wetting ridge was attributed to the vertical component of the water’s surface tension, which was high enough to lift the lubricant up.^47^ At the same time, the differences in the average CA between MS-LIS and LIPSS-LIS on both polymer materials became marginal and did not exceed 2°. The insignificance of surface topography on the wettability of as-prepared LIS clearly suggests that both surface topographies were fully encapsulated by the lubricant and most likely sharing the same spreading coefficient.

Next, the shedding behavior of water and Xanthan gum solution droplets on the plain, MS, LIPSS, MS-LIS, and LIPSS-LIS samples was investigated by measuring the minimum angle required to initiate their motion. Figure 4c,d shows CSAs of these liquids on all aforementioned surfaces of PC and COC substrates. Low droplet mobility was observed at CSAs ranging between 26° and 47° on the plain surfaces for all the tested liquids. Surprisingly, the textured surfaces of both thermoplastic materials did not facilitate any droplet movement even when the substrates were positioned vertically, i.e., at an inclination angle of 90°. In this case, the high adhesion forces induced from the water/solid contact resulted in pinning the droplets onto these surfaces. Instead, all LIS exhibited great shedding characteristics against water as the droplets slid on their surfaces at an average CSA below 13°. At the same time, CAH of water droplets was measured to vary within the ranges 8°–13° and 5°–8° on the MS-LIS and LIPSS-LIS samples, respectively. In reality, the infusion of lubricant into the textured surfaces dramatically lowered the contact line pinning and hence enabled the droplets to easily flow down their surfaces. However, it should be stated that only the LIPSS-LIS samples managed to repel both tested liquids at CSA smaller than 10°, indicating impressive shedding capabilities even against viscous liquids. Finally, because the endoscope lenses may be exposed to alkaline or acidic liquids, such as pancreatic and gastric juices, contained in the human body, CSAs for liquids with pH levels ranging from 1 to 9 were measured on all LIS onto the PC and COC substrates, as shown in Figures 4e and 4f, respectively. All the pH-adjusted water droplets were observed to start sliding on LIPSS-LIS of both polymer samples at CSA below 7°, while larger tilt angles were required to aid their movement on the MS-LIS ones, i.e., CSAs ranging from 8° up to 18°. The aforementioned results demonstrate the potential of LIPSS-LIS treated surfaces to shed almost any kind of biological fluid.

### 3.3 Durability of LIS under Shear Flow Conditions

**Figure 5:**
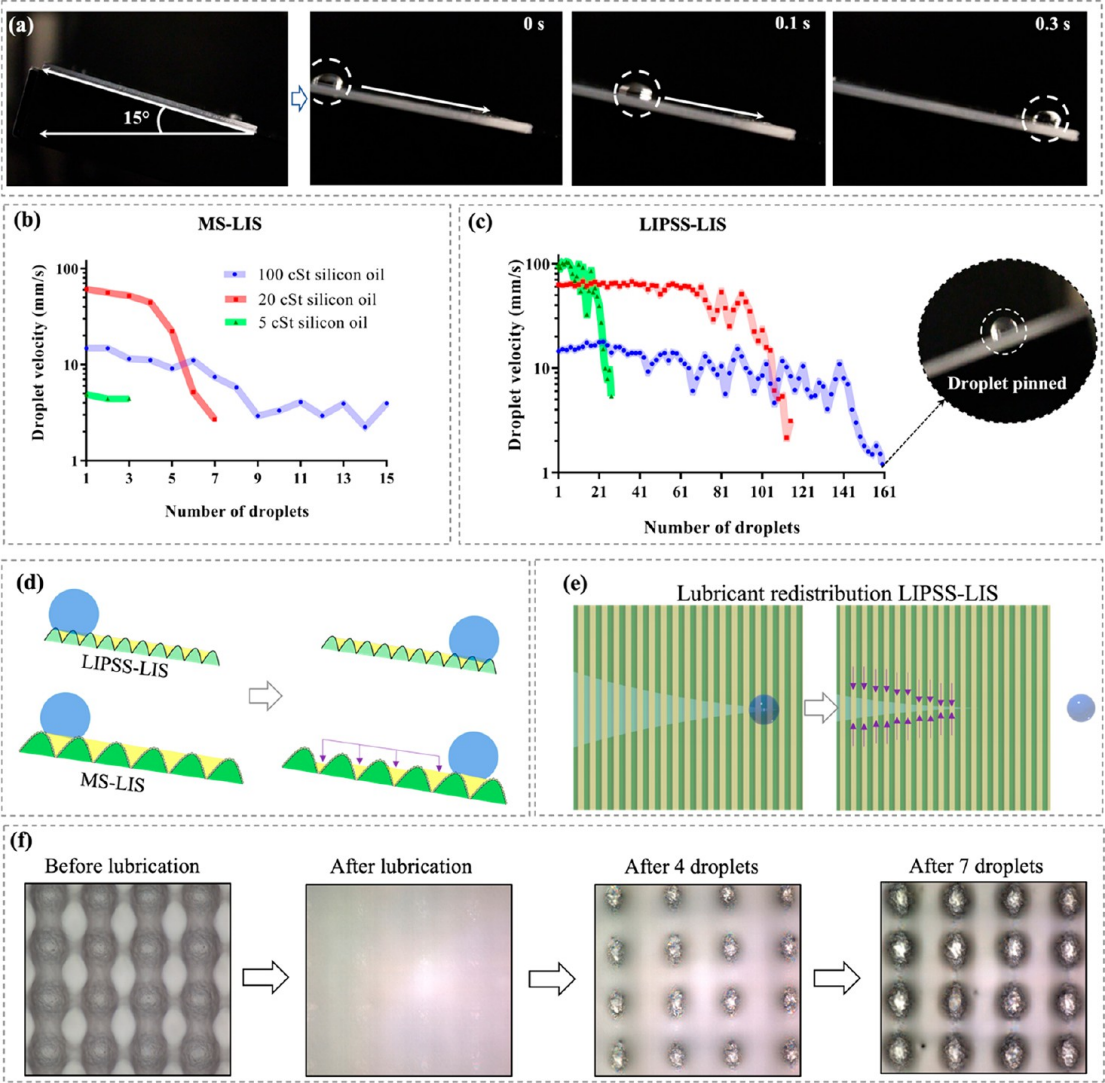
Shear-induced lubricant depletion: (a) Image showing a tilted substrate before the shear test and a water droplet sliding on the LIPSS-LIS sample impregnated with 5 cSt lubricant. (b, c) Shedding velocities as a function of the increasing number of water droplets deposited on the MS-LIS and LIPSS-LIS substrates for the investigated lubricant viscosities, respectively. Inset shows an image of water droplet pinned to the surface of the LIPSS-LIS sample after the lubricant depletion. (d) Illustration showing the lubricant depletion on LIPSS and MS topographies. (e) Illustration showing lubricant redistribution after a droplet leaving the LIPSS-LIS-treated surface. (f) Images depicting the loss of the infused lubricant from the MS-LIS samples.

As multiple single drops on LIS exert shear forces on them, thus they can cause severe depletion of the impregnated lubricant and subsequently lead to their failure. Therefore, the effect of such shear-induced lubricant depletion on the LIS shedding characteristics was investigated. In particular, LIS were tilted at an angle of 15° as depicted in Figure 5a and then subjected to water shear forces by repeatedly dispensing droplets at the same spot on their surfaces. Initially, all LIS exhibited low pinning forces regardless of the infused lubricant viscosity, and hence the water droplets shed off their surfaces by the gravity. An example of a water droplet sliding down LIPSS-LIS impregnated with the 5 cSt lubricant is given in Figure 5a. At the same time, the average shedding velocities of water droplets are plotted in Figures 5b and 5c as a function of the number of droplets deposited on the MS-LIS and LIPSS-LIS samples prepared with the lubricants of varying viscosity, respectively. In most cases, the droplet velocity decreased with the increase of lubricant viscosity because of the enhanced viscous dissipation at the liquid–lubricant interface. This trend agrees with previously reported studies.^48,49^ However, it should be noted that the lowest droplet sliding velocity was recorded on the MS-LIS sample when impregnated with the 5 cSt lubricant. This finding contradicts completely with the results obtained from the respective LIPSS-LIS one, on which the droplets exhibit the highest sliding velocity. It could be because there was not a stable entrapment of the low-viscosity lubricant within the microscale topography of the MS-LIS sample, and most likely it overflowed from the valleys when the surface was tilted.

After dripping multiple water droplets on the MS-LIS and LIPSS-LIS substrates prepared with varying lubricant viscosities, their slippery performance started to degrade. Especially, the droplet velocity decreased, and eventually the droplets were pinned to their surfaces. The degradation of the LIS response can be attributed to the gradual lubricant depletion. More specifically, the sliding droplets took away and/or displaced the lubricant from the impregnated structures due to the shear forces, resulting in a severe lubricant depletion along their flow track. However, LIPSS sustained the shear forces from a greater number of droplets than the MS ones. This is because the relatively strong nanocapillary forces manifested by the nanoscale ripples have rendered a longer lubricant storage capacity, as illustrated in Figure 5d, whereas the lubricant stored in the micrometer scale valleys of the MS topography was quickly depleted by the traveling train of droplets (see Figure 5d) which impacted the performance of MS-LIS. The images in Figure 5f also confirm that the infused lubricant was gradually removed by the sliding droplets and thus revealing the micropillar structures underneath the MS topographies. In addition to the strong capillary forces induced by the nanoscale structures,^50^ the droplets on the LIPSS-LIS samples were highly mobile. This is because LIS with nanostructured topographies alter the molecular orientation of water near the liquid–lubricant interface and have weaken the hydrogen-bonding interactions at the interface.^51^

At the same time, it can be noticed in Figure 5c that there are fluctuations in the shedding velocities of the droplets on LIPSS-LIS, irrespective of the lubricant viscosities. The lubricant redistribution might have caused a localized but temporarily finite replenishment of the lubricant layer,^52^ which can explain the fluctuations of droplet velocities. Especially, in the case of LIPSS, the depleted nanoscale ripple structures were swiftly replenished by the stored lubricant due to their strong capillary action as depicted in Figure 5e. Consequently, the velocity of the successive droplets increased until the next event of the lubricant depletion. This cyclic replenishment continues until all the stored lubricant had been completely depleted, and then the final pinning of the droplet occurred. Furthermore, it can also be noted that the lubricant depletion from the topographies was at a much slower rate when its viscosity increased. For instance, the samples impregnated with 100 cSt lubricant were able to retain their slippery properties much longer than those impregnated with 5 and 20 cSt ones. Overall, the LIPSS-LIS sample with 100 cSt lubricant viscosity exhibited the most robust and stable performance, and 160 droplets were required to lose completely its functionality.

### 3.4 Optical Analysis

**Figure 6:**
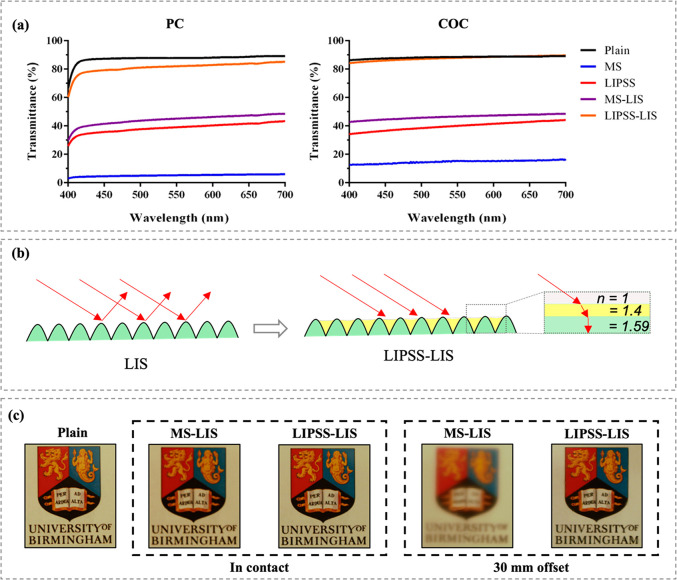
Analysis of optical properties: (a) the transmittance of the as-received, textured and lubricated surfaces of PC and COC, respectively; (b) schematic illustrations of the improved light transmittance after the lubricant infusion; (c) the captured images of a logo through the as-received, textured, and lubricated PC surfaces.

One of the most critical requirements for endoscope lenses is their high transparency. Therefore, the optical properties of all treated thermoplastic surfaces together with the as-received ones were assessed. As the refractive index was the same for all lubricants (*n* ∼ 1.4) used in this research irrespective of their viscosities, only the samples impregnated with 100 cSt lubricant were considered for these experiments. A comparison of the average transmittance obtained from the plain, MS, LIPSS, MS-LIS, and LIPSS-LIS substrates of PC and COC over the visible spectrum (400–700 nm) is provided in Figure 6a. As expected, the plain surfaces of both polymer materials showed an outstanding transparency of the visible light with peak transmittance values slightly below 90%. On the contrary, all the textured thermoplastic surfaces exhibited significantly low transmittance, indicating a translucent behavior. Such a dramatic loss of light was mainly attributed to its scattering by the relatively rough surfaces of the treated samples.^53,54^ Because such effect is typically more pronounced on surfaces with microscale roughness, the substrates with LIPSS appeared to be less opaque compared to the MS ones.

After the lubricant infusion into the textured thermoplastic surfaces, a substantial increase in transmittance was detected. Actually, the infused lubricant was able to minimize the negative effects of both light scattering and reflectance by smoothening the textured topographies and also reducing the large step change in the refractive index^55^ between air (*n* = 1) and the two thermoplastic materials (*n*_PC_ = 1.59 and *n*_COC_ = 1.4), as shown in Figure 6b. However, it should be stated that only the LIPSS-LIS samples exhibited an excellent transmittance of the visible light due to the relatively small surface area for a diffusive reflectance. In particular, the transmittance spectrum of the LIPSS-LIS sample almost matched that obtained on the plain COC one, whereas it dropped up to 10% on the treated PC surfaces within the visible spectrum. To compare further the transparency of the plain PC surface to that of LIPSS-LIS and MS-LIS, images of a colorful logo were captured with a camera when the samples were placed in contact with it and also with an offset distance of 30 mm, as shown in Figure 6c. In the former case, the logo was clearer through the LIPSS-LIS sample than the MS-LIS one, and it was also comparable with that taken through the plain surface. After positioning the substrates at a particular distance, the logo image appeared blurry through the MS-LIS sample due to light scattering by its microscale surface topography while its visibility was almost preserved through the LIPSS-LIS one. Therefore, only the LIPSS-LIS substrates were selected for further investigation in this research.

### 3.5 Antifouling Properties

#### 3.5.1 Antifogging and Blood Fouling Resistance

**Figure 7:**
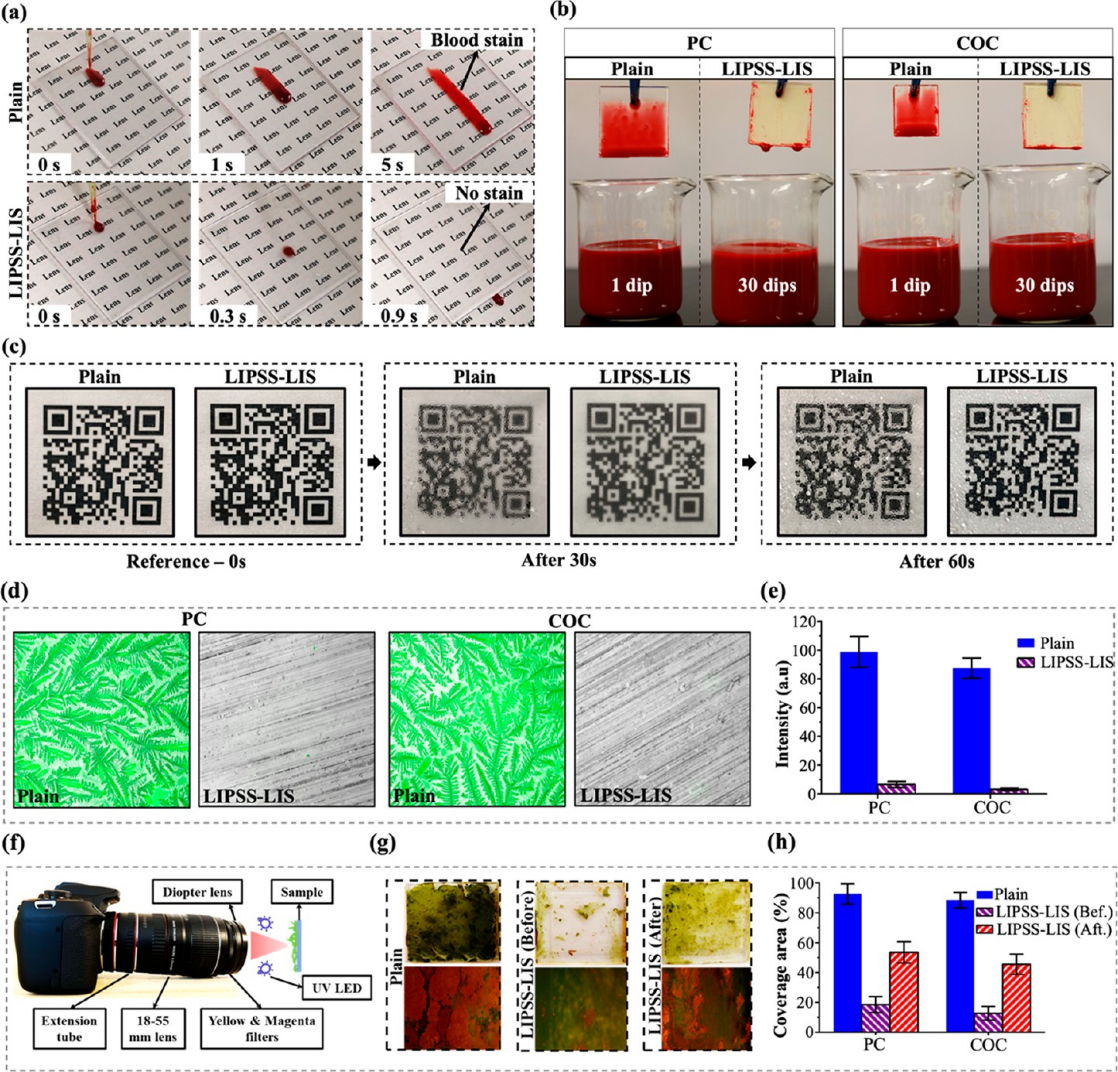
Evaluation of antifouling/antifogging performance: (a) Shedding behavior of 10 μL blood droplet on the PC plain and LIPSS-LIS samples. (b) Fouling resistance of PC and COC plain and LIPSS-LIS samples after the blood dipping test. It should be noted that the plain back sides of LIPSS-LIS samples were covered with a yellow colored tape to prevent their fouling by blood. (c) Sequential images taken from the PC plain and LIPSS-LIS samples after their exposure to hot vapor. (d) Fluorescence micrographs depicting the protein adsorption onto the PC and COC plain and LIPSS-LIS samples. (e) Plot showing the fluorescence intensity of adsorbed protein as measured on the PC and COC plain and LIPSS-LIS samples. (f) Experimental setup used for assessing and quantifying the adhesion of algae onto the surfaces. (g) Autofluorescence and optical images of algae adhesion onto the PC and COC plain surfaces together with their respective LIPSS-LIS samples before and after subjecting them to shear forces. (h) Area coverage with algae calculated on the samples.

The clarity of endoscope lenses can significantly be reduced due to fogging or blood fouling, resulting in impaired vision and consequently unnecessary disruptions during surgical procedures. Therefore, water condensation and blood adhesion experiments were performed to assess the functional response of LIPSS-LIS substrates. It should be stated that only the LIPSS-LIS samples prepared with the 100 cSt lubricant were subjected to testing because of their long-lasting slippery properties. Figure 7a shows the motion of a 10 μL blood droplet both on the plain and LIPSS-LIS samples of PC when tilted at an angle of ∼45°. The shedding behavior of such droplet on these surfaces can also be seen in Video S1. It is apparent that the droplet slowly slid along the plain surface and seemed to have sagged, leaving behind traces of blood. In contrast, a high blood droplet mobility was observed on the LIPSS-LIS sample without leaving any stain behind, which indicates an exceptional antifouling response. Similar results were obtained by the respective COC surfaces, too. Thereafter, the antifouling performance of LIPSS-LIS samples was evaluated by performing 30 dipping cycles into blood. Figure 7b shows that only a single dip was sufficient for the blood to adhere to the as-received PC and COC sheets. In contrast, the LIPSS-LIS samples of both polymer materials exhibited an excellent fouling resistance against blood because only a few microdroplets of blood were left behind on their surfaces after 30 blood dipping cycles (see Figure 7b).

Some fogging usually appears on lenses when the endoscopes enter the patient’s body due to the sudden change in humidity and temperature levels. Therefore, the antifogging properties of the LIPSS-LIS PC substrate were evaluated by using the so-called hot-vapor method, and they were compared with those obtained on the plain surface. More specifically, a printed QR code was attached to the back of these samples, and then they were placed vertically at a distance of roughly 50 mm above hot water (∼80 °C). Snapshots of the QR code through the plain and LIPSS-LIS samples were captured with a camera every 10 s and thus to test if they can retain their initial transparency. The sequential images in Figure 7c reveal that the hot vapor condensed on both surfaces and initially formed as small water droplets, exhibiting a “dropwise” condensation behavior. However, it should be stated that the nucleation rate of water droplets was more intense on the plain surface, while the low surface energy lubricant infused into the LIPSS-LIS sample delayed their formation. Such findings are consistent with a recent study, which investigated the droplet condensation on low and high surface energy materials.^56^ Within a period of 1 min, the small droplets appeared to gradually grow on the plain surface via a direct condensation and merged with the neighboring droplets to form larger ones. However, they were still not capable of shedding off the surface due to strong pinning forces. As a result, the large droplets formed on the surface scattered the transmitted light and distorted the vision through the plain sample (see the distorted image of the QR code in Figure 7c). In contrast, it can be seen in Figure 7c that some of the droplets condensed onto the LIPSS-LIS sample were removed due to some coalescence between them within less than 60 s, and thus the QR code could be clearly visible through the droplet-vacant regions. This shows that the low CAH of the LIPSS-LIS substrate led to high mobility of the condensed water droplets, which then promoted their fast coalescence with nearby droplets and led to droplets sweeping by gravitational force.

#### 3.5.2 Protein and Microalgae Fouling Resistance

In clinical environments, the usage of endoscopes in surgical procedure where they are in contact with blood increases the risk of thrombosis.^57^ The plasma protein adsorption on blood-contacting surfaces is the first event in the thrombus formation. Thus, the inhibition of protein adhesion to such surfaces is imperative in designing endoscopes, especially this applies to their lens surfaces. Therefore, the antiadhesive response of LIPSS-LIS when in contact with serum albumin has been investigated in this research as it is one of the most common blood proteins. Figures 7d and 7e show the composite fluorescent and bright field images of albumin fouling onto plain and LIPSS-LIS surfaces of PC and COC and their fluorescent intensity obtained, respectively. The serum albumin adhered to the plain PC and COC surfaces in a distinct dendritic pattern due to the NaCl present in the buffer after the thin film of a protein solution was evaporated, which is in accordance with the previous studies.^58^ At the same time, it is apparent that the protein adhesion is greatly inhibited on LIPSS-LIS when compared to the plain ones; especially there was more than 90% reduction. Therefore, the LIPSS-LIS treatment of endoscope lenses can decrease the risk of thrombus occurrence and thus reduces the probability of postsurgical complications in patients.

Microbial attachments to the endoscope surfaces, including lenses, during the surgical procedures can lead to endogenous infections. Therefore, there is a need to design the lens surfaces in such a way that they can inhibit the microbe’s adhesion. In our previous research, it was demonstrated that LIPSS and LIPSS-LIS exhibited antibacterial (*E. coli*) properties on polymer materials.^59^ The antifouling functionalities of thermoplastic LIPSS-LIS were investigated in this research under the influence of a microalgae *Chlorella vulgaris* (CV). Figure 7f shows the experimental setup used to investigate the CV’s attachment onto LIPSS-LIS-treated PC and COC substrates. As it is evident from both optical and fluorescent images in Figure 7g, CV favored the plain surfaces for settlement, while the LIPSS-LIS ones inhibited the CV’s adhesion to a larger extent. The image analysis results showed that LIPSS-LIS reduced the CV’s adhesion by almost 80% when compared to the plain surfaces (see Figure 7h). To assess further the durability of the LIPSS-LIS functional response, a set of substrates was initially attached to the wall of a glass container filled with water, and then the shear force was applied with a magnetic stirrer for 8 h. Such shear-induced LIPSS-LIS substrates were kept in culture baths as stated in section 2.7, and the fluorescent images were obtained to compare their performance with that of fresh LIPSS-LIS substrates. Impressively, even the shear-induced LIPSS-LIS have shown to reduce the CV’s adhesion by half when compared to the plain surfaces (see Figure 7g,h). This indicates that the strong nanoscale capillary forces of LIPSS resisted the lubricant depletion even under harsh condition, and their functionality was retained for longer.

### 3.6 Pilot Application

**Figure 8:**
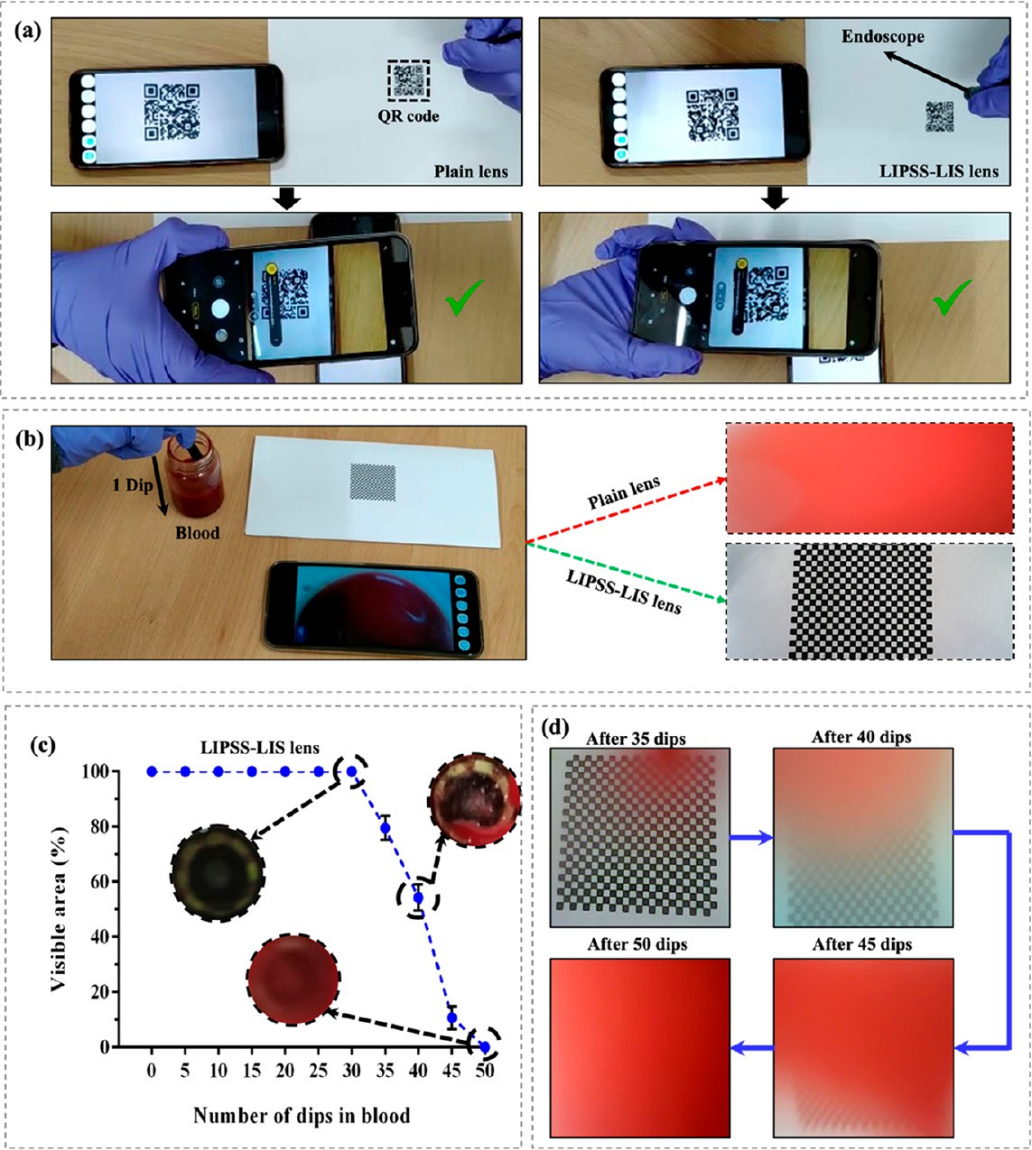
A pilot application of the LIPSS-LIS treated endoscope lenses: (a) Test procedure used to verify the vision performance after integrating the plain (left) and LIPSS-LIS (right) treated COC lenses into an endoscope device. (b) Blood dipping experimental setup and the visual fields retained by the plain and LIPSS-LIS treated COC lenes after a single dip. (c) Visible area in percentages of an endoscope with LIPSS-LIS lenses as a function of the dipping cycles number. Note: insets in (c) depict the surface of LIPSS-LIS lens after a certain number of dips; (d) sequential images showing the field of view after 35, 40, 45, and 50 dips, again for the LIPSS-LIS lens.

As proof of concept, a set of LIS lenses was integrated into an inspection endoscope device as stated in section 2.6, and then in vitro experiments were conducted to demonstrate its performance. In particular, LIPSS-LIS-treated COC lenses with 100 cSt lubricant were used in these tests as they fulfilled the criteria for slippery, durable, antifouling, and transparent surfaces. First, their vision performance was analyzed by reading a QR code. As shown in Figure 8a and Video S2, the QR code was imaged clearly through the LIPSS-LIS lens, and the time required to read it was less than 1 s, which was comparable with that achieved with the plain COC lens. In addition, the effectiveness of the LIPSS-LIS lens in maintaining a clear surgical field was investigated, too, by immersing the endoscope into blood once and then observing a printed boarding chess. From Figure 8b and Video S3, it is apparent that the visibility through the plain lens was immediately lost due to blood adhesion, while the LIPSS-LIS lens resisted blood fouling and retained its original clarity.

Finally, it was essential to test the LIPSS-LIS lens under harsh surgical conditions that might be encountered, e.g., when there are surgical complications with severe bleeding. Therefore, to validate its antifouling response under such conditions, the endoscope was repeatedly immersed into blood until the boarding chess could not be recognized anymore. In this regard, the visible area in percentages was measured every five blood dipping cycles, and the average results obtained from three LIPSS-LIS lenses are plotted in Figure 8c. From this plot, it is evident that the as-treated endoscope lenses did not exhibit any adhesion of blood after 30 dipping cycles (see the inset in Figure 8c), retaining 100% visibility. At the same time, only a small droplet of blood was formed on all the LIPSS-LIS lenses after 35 dips as shown in Figure 8d, leading to an average visibility decline of 20%. These findings confirm the superior blood fouling resistance of the LIPSS-LIS lenses in severe bleeding conditions and reinforce its performance advantages in minimizing vision losses in surgical endoscopic procedures. However, it should be noted that the antifouling performance of LIPSS-LIS lenses deteriorated dramatically afterward, i.e., after further immersion cycles into blood as evident by the sequential images in Figure 8d. For instance, a partial loss of visibility, i.e., an average vision loss of 46%, was observed after 40 dips. This was mostly due to the adherence of a larger blood droplet onto lens surfaces (see the inset in Figure 8c), and the visual field was less than 15% after 45 dips. At the end, the vision through the integrated camera was totally lost after 50 consecutive dipping cycles in blood (see Figure 8d), and they did not have any blood antifouling properties anymore (see the inset in Figure 8c).

## 4 Conclusions

Surface contamination and fouling of endoscope lenses by body fluids is a critical issue in surgical procedures, and therefore a cost-effective method for “imprinting” an antifouling functionality on them is proposed in this research. This is achieved by combining the strong capillary forces of LIPSS with the advantage of LIS in achieving long lasting antifouling properties without transparency losses. To fabricate lenses with such LIPSS-LIS treatments serially on thermoplastics, a process chain is proposed that combines synergistically the capabilities of a laser-based surface texturing of metallic masters with polymer micro/nano replication followed by lubricant infiltration onto the nanoscale surface ripples. Although a femtosecond laser was used to “imprint” LIPSS onto the masters, such topographies could be realized by less expensive pico- and nanosecond laser sources, which can further reduce the manufacturing cost. At the same time, micro injection molding technologies could be utilized instead of the lab-scale hot embossing to scale up the production of such LIPSS-LIS-treated thermoplastic lenses, as demonstrated in our previous research.^60^

Because the glass transition temperatures of PC and COC are less than the autoclave temperatures, such LIPSS-LIS thermoplastic lenses could be reused by employing standard sterilization procedures. Furthermore, it is worth noting that the LIPSS-LIS thermoplastic lenses produced with the proposed process chain are fully recyclable as no coatings are required to impart the antifouling functionalities. A simple washing step can remove the lubricant from the lenses for downstream recycling after their disposal. Furthermore, the functionality and durability of the proposed LIPSS-LIS treatments can be further improved by taking advantage of the LIPSS tunability. For example, instead of one-dimensional LIPSS employed in this research, two-dimensional triangular LIPSS^61^ can offer isotropic surface properties that can impart both the lubricant depletion of LIS and also their functional response.

## Supporting Information Available

Video S1: experimental video showing a 10 μL blood droplet sliding on the PC plain and LIPSS-LIS samples (MP4)
Video S2: visibility through an endoscope with COC plain and LIPSS-LIS lenses (MP4)
Video S3: blood fouling resistance of an endoscope integrated with LIPSS-LIS lenses (MP4)

## References

[ref1] Palvia V.; Gonzalez A.; Vigh R.; Anasti J. A randomized controlled trial comparing laparoscopic lens defogging techniques through simulation model. Gynecology and Minimally Invasive Therapy 2018, 7 (4), 156–160. 10.4103/GMIT.GMIT_39_18. 30306034 PMC6172873

[ref2] Quah G. S.; Eslick G. D.; Cox M. R. Laparoscopic appendicectomy is superior to open surgery for complicated appendicitis. Surgical Endoscopy 2019, 33 (7), 2072–2082. 10.1007/s00464-019-06746-6. 30868324

[ref3] Tatsuki H.; et al. A novel one-step lens cleaning device using air and water flow for endoscopic surgery. PLoS One 2018, 13 (7), e0200749 10.1371/journal.pone.0200749. 30020986 PMC6051665

[ref4] Manning T. G.; et al. Laparoscopic lens fogging: solving a common surgical problem in standard and robotic laparoscopes via a scientific model. Surgical Endoscopy 2018, 32 (3), 1600–1606. 10.1007/s00464-017-5772-x. 28791559

[ref5] Okuda H.; Okamoto J.; Takumi Y.; Kakehata S.; Muragaki Y. The iArmS Robotic Armrest Prolongs Endoscope Lens–Wiping Intervals in Endoscopic Sinus Surgery. Surgical Innovation 2020, 27 (5), 515–522. 10.1177/1553350620929864. 32603212

[ref6] Kreeft D.; Arkenbout E. A.; Henselmans P. W. J.; van Furth W. R.; Breedveld P. Review of Techniques to Achieve Optical Surface Cleanliness and Their Potential Application to Surgical Endoscopes. Surgical Innovation 2017, 24 (5), 509–527. 10.1177/1553350617708959. 28511635 PMC5603965

[ref7] Cassera M. A.; Goers T. A.; Spaun G. O.; Swanström L. L. Efficacy of Using a Novel Endoscopic Lens Cleaning Device: A Prospective Randomized Controlled Trial. Surgical Innovation 2011, 18 (2), 150–155. 10.1177/1553350611399297. 21343172

[ref8] Harawaza K.; Cousins B.; Roach P.; Fernandez A. Modification of the surface nanotopography of implant devices: A translational perspective. Materials Today Bio 2021, 12, 100152 10.1016/j.mtbio.2021.100152. PMC8554633 34746736

[ref9] Zhang S.; Zhou Y.; Zhang H.; Xiong Z.; To S. Advances in ultra-precision machining of micro-structured functional surfaces and their typical applications. International Journal of Machine Tools and Manufacture 2019, 142, 16–41. 10.1016/j.ijmachtools.2019.04.009.

[ref10] Guo H.; et al. A multifunctional anti-fog, antibacterial, and self-cleaning surface coating based on poly(NVP-co-MA). Chem. Eng. J. 2018, 351, 409–417. 10.1016/j.cej.2018.06.062.

[ref11] Topçu Kaya A. S.; Cengiz U. Fabrication and application of superhydrophilic antifog surface by sol-gel method. Prog. Org. Coat. 2019, 126, 75–82. 10.1016/j.porgcoat.2018.10.021.

[ref12] Bai S.; Li X.; Zhao Y.; Ren L.; Yuan X. Antifogging/Antibacterial Coatings Constructed by N-Hydroxyethylacrylamide and Quaternary Ammonium-Containing Copolymers. ACS Appl. Mater. Interfaces 2020, 12 (10), 12305–12316. 10.1021/acsami.9b21871. 32068389

[ref13] Geyer F.; et al. When and how self-cleaning of superhydrophobic surfaces works. Science Advances 2020, 6 (3), eaaw9727 10.1126/sciadv.aaw9727. 32010764 PMC6968945

[ref14] Gaddam A.; Sharma H.; Karkantonis T.; Dimov S. Anti-icing properties of femtosecond laser-induced nano and multiscale topographies. Appl. Surf. Sci. 2021, 552, 149443 10.1016/j.apsusc.2021.149443.

[ref15] Kim T.; Kwon S.; Lee J.; Lee J. S.; Kang S. A metallic anti-biofouling surface with a hierarchical topography containing nanostructures on curved micro-riblets. Microsyst. Nanoeng. 2022, 8 (1), 6 10.1038/s41378-021-00341-3. 35070350 PMC8743286

[ref16] Sharma H.; John K.; Gaddam A.; Navalkar A.; Maji S. K.; Agrawal A. A magnet-actuated biomimetic device for isolating biological entities in microwells. Sci. Rep. 2018, 8 (1), 12717 10.1038/s41598-018-31274-z. 30143719 PMC6109070

[ref17] Jalil S. A.; et al. Creating superhydrophobic and antibacterial surfaces on gold by femtosecond laser pulses. Appl. Surf. Sci. 2020, 506, 144952 10.1016/j.apsusc.2019.144952. 32184533 PMC7043332

[ref18] Ellinas K.; Dimitrakellis P.; Sarkiris P.; Gogolides E. A Review of Fabrication Methods, Properties and Applications of Superhydrophobic Metals. Processes 2021, 9 (4), 666 10.3390/pr9040666.

[ref19] Celik N.; Sahin F.; Ruzi M.; Yay M.; Unal E.; Onses M. S. Blood repellent superhydrophobic surfaces constructed from nanoparticle-free and biocompatible materials. Colloids Surf., B 2021, 205, 111864 10.1016/j.colsurfb.2021.111864. 34049000

[ref20] Li Z.; Nguyen B. L.; Cheng Y. C.; Xue J.; MacLaren G.; Yap C. H. Durable, flexible, superhydrophobic and blood-repelling surfaces for use in medical blood pumps. J. Mater. Chem. B 2018, 6 (39), 6225–6233. 10.1039/C8TB01547C. 32254613

[ref21] Eriksson M.; et al. Wetting Transition on Liquid-Repellent Surfaces Probed by Surface Force Measurements and Confocal Imaging. Langmuir 2019, 35 (41), 13275–13285. 10.1021/acs.langmuir.9b02368. 31547659

[ref22] Gaddam A.; Agrawal A.; Joshi S. S.; Thompson M. C. Utilization of Cavity Vortex To Delay the Wetting Transition in One-Dimensional Structured Microchannels. Langmuir 2015, 31 (49), 13373–13384. 10.1021/acs.langmuir.5b03666. 26598001

[ref23] Lin Y.; et al. Durable and robust transparent superhydrophobic glass surfaces fabricated by a femtosecond laser with exceptional water repellency and thermostability. J. Mater. Chem. A 2018, 6 (19), 9049–9056. 10.1039/C8TA01965G.

[ref24] Kunz C.; Müller F. A.; Gräf S. Multifunctional Hierarchical Surface Structures by Femtosecond Laser Processing. Materials 2018, 11 (5), 789 10.3390/ma11050789. PMC5978166 29757240

[ref25] Rapoport L.; Solomon B. R.; Varanasi K. K. Mobility of Yield Stress Fluids on Lubricant-Impregnated Surfaces. ACS Appl. Mater. Interfaces 2019, 11 (17), 16123–16129. 10.1021/acsami.8b21478. 31008574

[ref26] Wong T.-S.; et al. Bioinspired self-repairing slippery surfaces with pressure-stable omniphobicity. Nature 2011, 477 (7365), 443–447. 10.1038/nature10447. 21938066

[ref27] Badv M.; Imani S. M.; Weitz J. I.; Didar T. F. Lubricant-Infused Surfaces with Built-In Functional Biomolecules Exhibit Simultaneous Repellency and Tunable Cell Adhesion. ACS Nano 2018, 12 (11), 10890–10902. 10.1021/acsnano.8b03938. 30352507

[ref28] Yuan S.; Luan S.; Yan S.; Shi H.; Yin J. Facile Fabrication of Lubricant-Infused Wrinkling Surface for Preventing Thrombus Formation and Infection. ACS Appl. Mater. Interfaces 2015, 7 (34), 19466–19473. 10.1021/acsami.5b05865. 26268298

[ref29] Sharma H.; Gaddam A.; Agrawal A.; Joshi S. S. Slip flow through microchannels with lubricant-infused bi-dimensional textured surfaces. Microfluid. Nanofluid. 2019, 23 (2), 28 10.1007/s10404-019-2197-y.

[ref30] Gurav A. B.; Shi H.; Duan M.; Pang X.; Li X. Highly transparent, hot water and scratch resistant, lubricant-infused slippery surfaces developed from a mechanically-weak superhydrophobic coating. Chem. Eng. J. 2021, 416, 127809 10.1016/j.cej.2020.127809.

[ref31] Zhang M.; et al. Ultra-transparent slippery surface. Smart Materials in Medicine 2021, 2, 38–45. 10.1016/j.smaim.2020.10.001.

[ref32] Zhang M.; Liu Q.; Liu J.; Yu J.; Wang J. Self-healing liquid-infused surfaces with high transparency for optical devices. MRS Commun. 2019, 9 (1), 92–98. 10.1557/mrc.2018.241.

[ref33] Zhang P.; Chen H.; Zhang L.; Ran T.; Zhang D. Transparent self-cleaning lubricant-infused surfaces made with large-area breath figure patterns. Appl. Surf. Sci. 2015, 355, 1083–1090. 10.1016/j.apsusc.2015.07.159.

[ref34] Manabe K.; Kyung K.-H.; Shiratori S. Biocompatible Slippery Fluid-Infused Films Composed of Chitosan and Alginate via Layer-by-Layer Self-Assembly and Their Antithrombogenicity. ACS Appl. Mater. Interfaces 2015, 7 (8), 4763–4771. 10.1021/am508393n. 25646977

[ref35] Liu M.; Hou Y.; Li J.; Tie L.; Guo Z. Transparent slippery liquid-infused nanoparticulate coatings. Chem. Eng. J. 2018, 337, 462–470. 10.1016/j.cej.2017.12.118.

[ref36] Nishioka S.; et al. Facile design of plant-oil-infused fine surface asperity for transparent blood-repelling endoscope lens. RSC Adv. 2016, 6 (53), 47579–47587. 10.1039/C6RA08390K.

[ref37] Sunny S.; et al. Transparent antifouling material for improved operative field visibility in endoscopy. Proc. Natl. Acad. Sci. U. S. A. 2016, 113 (42), 11676 10.1073/pnas.1605272113. 27688761 PMC5081611

[ref38] Tenjimbayashi M. In Situ Formation of Slippery-Liquid-Infused Nanofibrous Surface for a Transparent Antifouling Endoscope Lens. ACS Biomater. Sci. Eng. 2018, 4 (5), 1871–1879. 10.1021/acsbiomaterials.8b00134. 33445342

[ref39] Lee Y.; et al. Lubricant-infused directly engraved nano-microstructures for mechanically durable endoscope lens with anti-biofouling and anti-fogging properties. Sci. Rep. 2020, 10 (1), 17454. 33060752 10.1038/s41598-020-74517-8 PMC7566624

[ref40] Sunderland E. M.; Hu X. C.; Dassuncao C.; Tokranov A. K.; Wagner C. C.; Allen J. G. A review of the pathways of human exposure to poly- and perfluoroalkyl substances (PFASs) and present understanding of health effects. Journal of Exposure Science & Environmental Epidemiology 2019, 29 (2), 131–147. 10.1038/s41370-018-0094-1. 30470793 PMC6380916

[ref41] Han J.; et al. Chemical Aspects of Human and Environmental Overload with Fluorine. Chem. Rev. 2021, 121 (8), 4678–4742. 10.1021/acs.chemrev.0c01263. 33723999 PMC8945431

[ref42] Karkantonis T.; Gaddam A.; See T. L.; Joshi S. S.; Dimov S. Femtosecond laser-induced sub-micron and multi-scale topographies for durable lubricant impregnated surfaces for food packaging applications. Surf. Coat. Technol. 2020, 399, 126166 10.1016/j.surfcoat.2020.126166.

[ref43] Ozkan E.; et al. Bioinspired ultra-low fouling coatings on medical devices to prevent device-associated infections and thrombosis. J. Colloid Interface Sci. 2022, 608, 1015–1024. 10.1016/j.jcis.2021.09.183. 34785450 PMC8665144

[ref44] et al. Re-evaluation of dimethyl polysiloxane (E 900) as a food additive. EFSA Journal 2020, 18 (5), e06107. 10.2903/j.efsa.2020.6107 PMC10464691 37649521

[ref45] Gaddam A.; Sharma H.; Ahuja R.; Dimov S.; Joshi S.; Agrawal A. Hydrodynamic drag reduction of shear-thinning liquids in superhydrophobic textured microchannels. Microfluid. Nanofluid. 2021, 25 (9), 73 10.1007/s10404-021-02470-7.

[ref46] Baumli P.; et al. The challenge of lubricant-replenishment on lubricant-impregnated surfaces. Adv. Colloid Interface Sci. 2021, 287, 102329 10.1016/j.cis.2020.102329. 33302056

[ref47] Sartori P.; et al. Motion of Newtonian drops deposited on liquid-impregnated surfaces induced by vertical vibrations. J. Fluid Mech. 2019, 876, R4 10.1017/jfm.2019.600.

[ref48] Keiser A.; Keiser L.; Clanet C.; Quéré D. Drop friction on liquid-infused materials. Soft Matter 2017, 13 (39), 6981–6987. 10.1039/C7SM01226H. 28933489

[ref49] Adera S.; Alvarenga J.; Shneidman A. V.; Zhang C. T.; Davitt A.; Aizenberg J. Depletion of Lubricant from Nanostructured Oil-Infused Surfaces by Pendant Condensate Droplets. ACS Nano 2020, 14 (7), 8024–8035. 10.1021/acsnano.9b10184. 32490664

[ref50] Wong W. S. Y.; Hegner K. I.; Donadei V.; Hauer L.; Naga A.; Vollmer D. Capillary Balancing: Designing Frost-Resistant Lubricant-Infused Surfaces. Nano Lett. 2020, 20 (12), 8508–8515. 10.1021/acs.nanolett.0c02956. 33206541 PMC7735743

[ref51] Zhang C.; Adera S.; Aizenberg J.; Chen Z. Why Are Water Droplets Highly Mobile on Nanostructured Oil-Impregnated Surfaces?. ACS Appl. Mater. Interfaces 2021, 13 (13), 15901–15909. 10.1021/acsami.1c01649. 33754694

[ref52] Jacobi I.; Wexler J. S.; Stone H. A. Overflow cascades in liquid-infused substrates. Phys. Fluids 2015, 27 (8), 082101 10.1063/1.4927538.

[ref53] Zhang M.; et al. Highly transparent and robust slippery lubricant-infused porous surfaces with anti-icing and anti-fouling performances. J. Alloys Compd. 2019, 803, 51–60. 10.1016/j.jallcom.2019.06.241.

[ref54] Ahmad S.; Sharma H.; Agrawal A.; Joshi S. S. Light Harvesting Using Biomimetic Micro-textured Transparent Films for Photovoltaic Applications. Transactions of the Indian National Academy of Engineering 2021, 6 (3), 775–785. 10.1007/s41403-021-00244-9.

[ref55] Wang Z.; Guo Z. Biomimetic self-slippery and transferable transparent lubricant-infused functional surfaces. Nanoscale 2018, 10 (42), 19879–19889. 10.1039/C8NR07608A. 30335109

[ref56] Cha H.; et al. Dropwise condensation on solid hydrophilic surfaces. Science Advances 2020, 6 (2), eaax0746 10.1126/sciadv.aax0746. 31950076 PMC6954056

[ref57] Venkatachalapathy S. V.; Evans G.; Muller A. F. Endoscopy and the Risk of Venous Thromboembolism: A Case-Control Study. Endosc Int. Open 2014, 2 (01), E2–E5. 10.1055/s-0034-1365277. 26134608 PMC4476430

[ref58] Shiba K.; Honma T.; Minamisawa T.; Nishiguchi K.; Noda T. Distinct macroscopic structures developed from solutions of chemical compounds and periodic proteins. EMBO reports 2003, 4 (2), 148–153. 10.1038/sj.embor.embor737. 12612603 PMC1315835

[ref59] Siddiquie R. Y.; Gaddam A.; Agrawal A.; Dimov S. S.; Joshi S. S. Anti-Biofouling Properties of Femtosecond Laser-Induced Submicron Topographies on Elastomeric Surfaces. Langmuir 2020, 36 (19), 5349–5358. 10.1021/acs.langmuir.0c00753. 32343580

[ref60] Baruffi F.; et al. Correlating nano-scale surface replication accuracy and cavity temperature in micro-injection moulding using in-line process control and high-speed thermal imaging. Journal of Manufacturing Processes 2019, 47, 367–381. 10.1016/j.jmapro.2019.08.017.

[ref61] Romano J.-M.; Garcia-Giron A.; Penchev P.; Dimov S. Triangular laser-induced submicron textures for functionalising stainless steel surfaces. Appl. Surf. Sci. 2018, 440, 162–169. 10.1016/j.apsusc.2018.01.086.

